# Commercial Baby Foods Aimed at Children up to 36 Months: Are They a Matter of Concern?

**DOI:** 10.3390/foods11101424

**Published:** 2022-05-13

**Authors:** Mariana Santos, Filipa Matias, Isabel Loureiro, Ana Isabel Rito, Isabel Castanheira, Alexandra Bento, Ricardo Assunção

**Affiliations:** 1 Food and Nutrition Department, National Health Institute Dr Ricardo Jorge, Av. Padre Cruz, 1649-016 Lisbon, Portugal; filipa.matias@insa.min-saude.pt (F.M.); ana.rito@insa.min-saude.pt (A.I.R.); isabel.castanheira@insa.min-saude.pt (I.C.); alexandra.bento@insa.min-saude.pt (A.B.); rassuncao@egasmoniz.edu.pt (R.A.); 2 NOVA National School of Public Health, NOVA University of Lisbon, Avenida Padre Cruz, 1600-560 Lisbon, Portugal; 3 NOVA National School of Public Health, Public Health Research Center (CISP), Comprehensive Health Research Center, NOVA University of Lisbon, Avenida Padre Cruz, 1600-560 Lisbon, Portugal; isalou@ensp.unl.pt; 4 Center for Studies and Research in Social Dynamics and Health (CEIDSS), Av. Padre Cruz, 1649-016 Lisbon, Portugal; 5 MARE-NOVA-Marine and Environmental Sciences Centre, Department of Environmental Sciences and Engineering, NOVA School of Science and Technology (FCT NOVA) (FCT/UNL), 2829-516 Caparica, Portugal; 6 CESAM, Centre for Environmental and Marine Studies, University of Aveiro, Campus Universitário de Santiago, 3810-193 Aveiro, Portugal; 7 IUEM–Instituto Universitário Egas Moniz, Egas Moniz-Cooperativa de Ensino Superior, 2829-511 Caparica, Portugal

**Keywords:** nutrient profile model, commercially available complementary foods, sugars, ultra-processed foods, infants and young children

## Abstract

Proper nutrition in infancy and early childhood is crucial to ensuring optimal child development, growth, and better health outcomes later in life. The nutrient profile model proposed by WHO/Europe aims to assess the nutritional quality and promotional/marketing aspects of commercial baby foods aimed at children up to 36 months. We used commercial data from 191 baby foods collected between March 2021 and July 2021, from eight supermarket chains in the Lisbon Metropolitan area. According to the model specifications and the NOVA classification system, we assessed the nutritional quality and promotion aspects and the degree of processing, respectively. The presence of at least one sugar-contributing ingredient was found in 34.0% of the products; 13.9% of products listed sugars and 15.0% listed fruit juices or concentrates as an ingredient. The claim “No added sugar” was present in 69.6% of products. Only 35.1% of products comply with all the nutritional requirements of the model. Concerning processing classification, 61.8% of products were ultra-processed, and about 57.0% were indicated for children < 12 months. These findings reinforce the importance of implementing measures to ensure that commercial foods for infants are marketed appropriately and to promote foods with a lower degree of processing.

## 1. Introduction

In infancy, adequate nutrition is a key factor for normal growth and development, helps the establishment of taste preferences, and may have implications for health throughout life [1]. Food preferences begin to take shape during fetal development and continue to change throughout life, influenced by biological, social, and environmental factors [2]. These preferences are fundamental of food choice and therefore diet quality.

The promotion of exclusive breastfeeding for six months is the gold standard for infant feeding and a global public health recommendation [3]. Infants should receive safe and nutritionally adequate complementary foods from 6 months of age, keeping breastfeeding until 2 years of age or older [4]. The role of these products in an appropriate complementary feeding has been discussed, driven by concerns related to their nutritional content and potentially problematic marketing strategies associated with the promotion of these products [5].

In 2016, the WHO guidance on ending the inappropriate promotion of foods for infants and young children (IYC) was approved to endorse countries to take action on this issue [6,7]. To implement this guidance, a Nutrient Profile Model (NPM) for commercially available complementary foods (CACFs) for infants under the age of 36 months was developed to drive the decisions regarding the identification of foods that are inappropriate for promotion, particularly reducing the intake of salt and free sugars [8].

Currently, Directive 2006/125/EC does not specify the total sugar content but sets out the amount of added sugar, such as sucrose, fructose, syrups, and honey, that is allowed in processed cereal-based foods. However, as this information is not included on the packaging, it is not possible to determine the amount of these or the total added (or free) sugar in products [9].

Children’s diets are generally characterised by low fruit and vegetable consumption and an excess of products that contain high levels of sugar, saturated fat, and sodium [10]. In particular, sugar consumption among children has been a cause of concern worldwide, as it is higher than children’s recommended daily limit. 

The WHO recommends as a “strong recommendation” reducing the intake of free sugars to less than 10% of total energy intake as well as a “conditional recommendation” to further reduce free sugars to below 5% [11].

Published data show that this reducing the intake of free sugars to less than 10% of total energy intake is not met in most countries; for example, in several European countries, total sugars ranged between 15 and 21% of energy intake and between 16 and 26% in adults and children, respectively [12]. Added sugars provide 7 to 11% of total energy intake in adults and contributes with a higher proportion of children’s energy intake (11 to 17%) [12]. In Portugal, the National Food, Nutrition, and Physical Activity Survey reported that IYC (<5 years) had the highest energy contribution from total sugars (28%) [13]. In addition, evidence is growing that some CACFs for infants (also referred as baby foods) contain considerable high amounts of sugar [14,15,16,17,18]. 

In the first two years of life, the introduction of complementary food represents an opportunity for infants to achieve long-term healthy dietary patterns [19]. Recent studies have found that products containing ingredients such as sugar, salt, fat, and artificial colors or preservatives and produced from constituents derived from foods, also known as ultra-processed (UP) foods, comprise an increasing percentage of children’s diets [20]. In the Belgian population, about 30% of daily energy intake is from UP foods, and young children consume the largest proportion of their daily energy intake from UP foods [21]. Other studies, such as from the U.S. and Canada, also found that UP foods represent more than 50% of the daily energy intake [22,23].

Food processing increases nutrient availability and food quality and plays a key role in attaining food and nutrition security [24]. UP foods are usually low in fiber, protein, vitamins, and minerals and high in added sugar, trans-fat, sodium, and refined starch [23]. The consumption of UP foods during childhood has also been linked to obesity and cardiometabolic risk factors in children and adults [25,26] as well as an increased mortality risk among adults [27,28]. 

According to a Sadler et al. review, there are a great number of food classification systems based on food processing. Most of the classification systems are proposed by expressing concerns about the food transition to industrially made products and an associated increase in chronic disease [29]. 

The NOVA classification system developed by Monteiro et al. has been extensively applied in several studies of diet quality and health outcomes, particularly obesity [30]. This system classifies foods and food products into four categories according to the degree of processing, comprising unprocessed and minimally processed foods (e.g., vegetables and fresh fruit), processed culinary ingredients (e.g., honey and sugar), processed foods (e.g., fruits in syrup and vegetables in brine), and UP foods (e.g., packaged soups and chicken nuggets) [31]. 

Several studies have evaluated the consumption of UP foods in paediatric populations [19,32]; however, there is still limited research on the assessment of CACFs marketed for IYC under 36 months according to the NOVA classification system [17,18,33].

In 2019, the National Health Authority (Directorate General of Health (DGS)) developed a Nutrient Profile Model (PT_NPM) based on the WHO nutrient profile model with some adaptions. This model was created to limit food promotion/marketing to children between 36 months and 16 years old [34]. As adequate nutrition in infancy and early childhood is crucial to prevent all forms of malnutrition and diet-related non-communicable diseases, effective action is then needed for ending the inappropriate promotion of foods for infants and young children under 36 months [7]. The implementation of the Nutrient Profile Model (NPM) developed by the WHO Regional Office for Europe (WHO/Europe) to guide decisions on the promotion and nutritional quality of baby foods will be under the scope of the Integrated Strategy for the Promotion of Healthy Eating (EIPAS) in Portugal [35].

The present study aims to assess the nutritional quality and promotion (labelling requirements, visual information on labels and type of statements) of products marketed for IYC under 36 months available on the Portuguese market considering the following aspects:

(1) Agreement with the compositional criteria and labelling requirements of the Nutrient Profile Model (NPM) for commercial baby foods aimed at children up 36 months;
(2) Level of processing as defined by the NOVA classification system.

## 2. Materials and Methods

### 2.1. Data Collection

This study used a cross-sectional design and a convenience sample of 191 products targeted to infants under 36 months of age (CACFs), collected between March 2021 and July 2021, from eight supermarket chains in the Lisbon Metropolitan area, which holds almost 80% of the market share in Portugal. Following the method of a previously published study [15], nutritional and packaging information and information about the ingredient list from the products in-store or the supermarkets’ websites were collected and photographed. 

A Microsoft Excel spreadsheet was developed to record information on the different variables studied, namely basic information (brand name, product name, product category); packaging information (recommended age, serving size, nutrition-related messages, composition claims, and health claims); nutritional content (energy (kJ/kcal), protein (g), total fat (g), saturated fatty acids, carbohydrates (g), total sugars (g), and sodium (mg)); and visual information (e.g., cartoons, pictures of infants/young children). All the nutritional values have been expressed per 100 g product. 

### 2.2. Product Categorization

**Figure 1:**
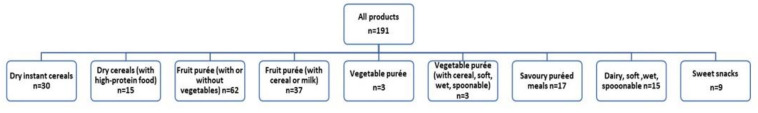
Sampling and Product categorization according to the study.

CACFs were categorized based on the adopted NPM classification developed by the WHO/Europe [8] and presented in Figure 1.

CACFs were also categorized according to their processing level as minimally processed (MP), processed, or UP foods based on the NOVA classification system [31] using the photographs of the ingredients list. Foods whose ingredients list only presented unprocessed foods (e.g., fruit and vegetables) were categorized as minimally processed. Products with ingredients such as salt, sugar, and fats were classified as processed. For the classification of UP products, the use of industrial techniques, such as extrusion, hydrogenation, and carbohydrate modifications, and the ingredient list was taken into account. Food products where the ingredient list contained additives, such as emulsifiers, and flavourings, were categorised as UP [16,18]. Information on the coded variables is provided in Appendix A (Table A1). 

### 2.3. Nutritional Composition and Labelling Requirements Evaluation

Nutrient composition, labelling requirements, and promotional restrictions were evaluated according to the adopted criteria of NPM for CACFs to identify products appropriate for promotion for IYC up to 36 months [8].

Results for energy, protein, total sugars, total fat, saturated fatty acids, and sodium are presented on g (or mg) per 100 g. The energy density is shown on a kcal per 100 g basis. In addition, the nutrient values in g per 100 kcal were calculated to compare with dry products that have to be reconstituted before consumption and ready-to-eat products.

The presence of added sugars was identified from the back-of-pack ingredient list. For the present study, added sugar was classified as fruit juice whether whole, concentrated, or powdered; sugar; sucrose; dextrose; fructose; maltose; any syrup; honey; barley malt/malted barley/malt extract; molasses; and artificial or natural zero/low-calorie sweeteners, in line with the WHO definition of free sugar [11]. This is an extension of the European Food Safety Authority (EFSA) definition of added sugars [36] by additionally including fruit juice (and its derivatives) and honey.

### 2.4. CACFs Classification into Target Age Groups

CACFs were divided into four groups, based on the developmental milestones for feeding and the minimum age specified on the food label [37]. Group 1 includes foods for infants from 4 months to <6 months, Group 2 from 6 months to <8 months, Group 3 from 8 months to <12 months, and Group 4 from 12 months or older. 

### 2.5. Data Analysis

Data were processed with the Statistical Package for Social Sciences software and included frequency distribution, descriptive statistics, and correlation (IBM SPSS Statistics, Version 28.0, IBM Corp., Chicago, IL, USA) [38].

Descriptive statistics had been used to report the frequency of CACFs in each food category, food processing (NOVA classification), age, and claims. 

Tests for normality on the nutritional values of foods were performed with the Kolmogorov–Smirnov test and revealed that data were not normally distributed; therefore, non-parametric testing was conducted.

The categorical variables were reported as relative and absolute frequencies. Median, standard deviation (SD), and P_25_ and P_50_ values for nutritional information were calculated from the labels. Pearson’s chi-square test (χ^2^ test) was employed to assess the associations between two variables. Kruskal–Wallis non-parametric test with multiple pairwise comparisons was performed to test for differences in levels of nutrients between food categories and food processing (NOVA classification). The Bonferroni correction was applied. For a *p*-value less than 0.05, the result was considered statistically significant and highly statistically significant if the *p*-value was less than 0.01. Results are shown in tables and box plot diagrams.

## 3. Results

### 3.1. General Characteristics

Table 1 provides information on the number of products by categories for which information was collected, respectively.

Of the 191 CACFs identified in this study, the most abundant categories of CACFs were fruit purée (with/without vegetables) (*n* = 62, 32.5%), fruit purée with cereal/milk (*n* = 37, 19.4%), and dry instant cereals (*n* = 30, 15.7%).

The majority (*n* = 177, 92.7%) of CACFs were indicated for children under 12 months of age (Groups 1–3). As shown in Table 1, 12.6% of CACFs were intended to be consumed by Group 1 (4 to <6 months) children, 61.3% for Group 2 (6 to <8 months) children, 18.8% for Group 3 (8 to <12 months) children, and 7.3% for Group 4 (≥ 12 months) children.

### 3.2. Nutritional Composition

Table 2 summarizes the nutritional composition of the CACFs considered in the study.

Assessment of the nutritional composition per 100 g, the categories dry instant cereals, fruit purée (with/without vegetables), fruit purée with cereal/milk, and vegetables with cereal, soft, wet, spoonable presented significantly lower values for sodium (*p* < 0.001). The lower medium energy content was found in vegetable purée (105 kJ/100 g) and vegetables with cereals/milk products (223 kJ/100 g), and the highest content was found in sweet snacks (1810 kJ/100 g). Dry cereals with high protein, dairy, soft, wet, spoonable and sweet snacks showed the highest median saturated fatty acids content (Table 2). 

**Figure 2:**
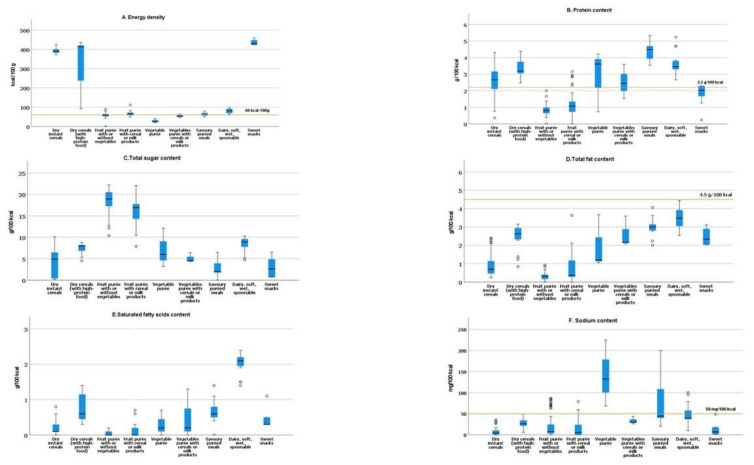
(**A**) Energy density of food products. The horizontal yellow line at 60 kcal/100 g indicates the minimum energy density adopted by the NPM of the WHO/Europe for several CACFs categories. (**B**) Protein content of food products. The line at 2.2 g/100 kcal indicates a lower limit adopted by the NPM of the WHO/Europe for several CACFs categories. (**C**) Total sugar content of food products; (**D**) total fat content of food products; (**E**) saturated fatty acids content of food products. The yellow line at 4.5 g/100 kcal indicates an upper limit for total fat adopted by the NPM of the WHO/Europe for some CACFs categories; (**F**) sodium content of food products. The yellow line at 50 mg/100 kcal indicates the upper limit for sodium adopted by the NPM of the WHO/Europe for some CACFs categories.

Analysing the energy density (kcal/100 g) by food category, Figure 2A shows that some products, such as the case of vegetable purée and vegetables with cereals/milk products, provide a lower energy density, less than 60 kcal/100 g.

Regarding protein content (Figure 2B), our study shows that most of the products exceed the adopted lower limit of total protein (2.2 g/100 kcal), proposed by the NPM of the WHO/Europe, for several CACFs categories.

The total sugar content ranged from 0 g/100 kcal to 22.0 g/100 kcal (Figure 2C). The highest content of total sugars was observed for dry cereals with high protein, dry instant cereals, and sweet snacks (27.2, 20.0 and 17.2 g per 100 g of product, respectively). The average energy contribution from total sugars varied between 8.1% (spoonable meals) to 75.7% fruit purée (with/without vegetables). 

**Figure 3:**
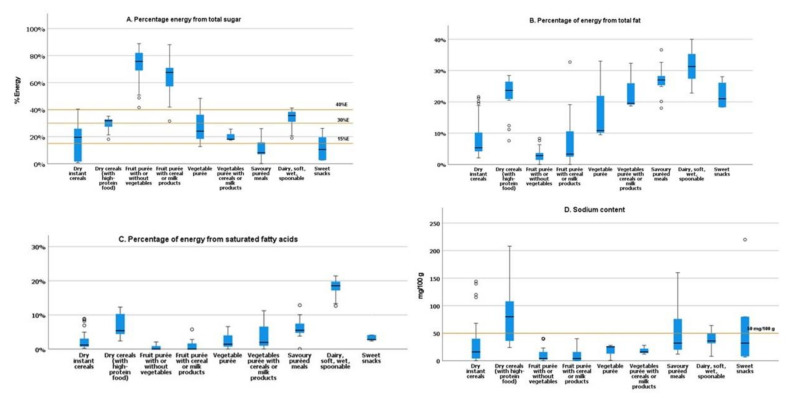
(**A**) Percentage energy from total sugar of food products; the yellow lines at 15%, 30%, and 40% indicate the adopted percentage of calories from total sugar to have a flag on the front-of-pack label by the NPM of the WHO/Europe for several CACFs categories. (**B**) Percentage energy from total fat of food products; (**C**) percentage energy from total saturated fatty acids of food products; (**D**) sodium content (mg/100 g) of food products. The yellow line at 50 mg/100 g indicates the upper limit for sodium adopted by the NPM of the WHO/Europe for some CACFs categories.

For all the fruit purée (with/without vegetables) (*n* = 62), fruit with cereal/milk products (*n* = 37), and half of the dry instant cereals with high protein (*n* = 8), more than 30% of calories come from total sugar, and around 30% (*n* = 5) of the savoury puréed meals have more than 15% of calories from sugars (Figure 3A). 

Concerning the energy contribution from total sugar (Figure 3A), the fruit purée with/without vegetables (*n* = 62) and the fruit with cereal/milk products (*n* = 37) tend to contain a high sugar content, with > 30% of calories from sugars. 

For total fat content, a considerable degree of variation in different categories was verified (Figure 2D). The highest percentage of energy from total fat was observed in the dairy, soft, wet, spoonable category (Figure 3B).

The highest medium level of saturated fatty acids per 100 kcal was observed in the category dairy, soft, wet, spoonable (2.1 g/100 kcal) (Figure 2E). For most of the products, the average saturated fatty acids content was less than or equal to 10% energy from saturated fat (Figure 3C).

For sodium content, the NPM for CACFs proposed by the WHO/Europe comprises maximum limits for sodium, 50 mg/100 kcal (and 50 mg/100 g) or 100 mg/100 kcal (and 100 mg/100 g), when a product contains cheese. 

Considering the maximum limit adopted by the NPM for sodium 50 mg/100 g, we observed some products in the categories savoury puréed meals; dairy, soft, wet, spoonable; and sweet snacks exceeding the adopted limit (Figure 3D) and would require reformulation to reduce the sodium levels. 

### 3.3. Compliance with the NPM Criteria

#### 3.3.1. Compositional Criteria

Nutrition information was assessed according to the individual requirements of the NPM proposed by the WHO/Europe. From the 191 CACFs under study, only 35.1% (*n* = 67) have been identified as appropriate for children up to 36 months, as they meet all the compositional criteria of the model. 

Regarding protein and fat content, all products meet the recommended levels. Around 70% (*n* = 134) had no added sugars/sweeteners, and 77.5% (*n* = 148) had lower than recommended sodium levels. An energy threshold of 60 kcal/100 g (minimum) is proposed by the model for products in the fruit/vegetables purées and dairy categories. This study found that 40.8% (*n* = 78) of products from these categories provide higher calories than the recommended.

#### 3.3.2. Labelling Requirements

Most of the CACFs carried a composition/nutrition claim (96.9%), with health claims present on 9.0% of products. Some visual aspects of product labels have been correlated with inappropriate promotion, while 39.0% (*n* = 74) of the products carried cartoon images (Table 3). 

On the other hand, 69.6% (*n* = 133) of the total products under study had “No added sugar” claim, with fruit purée with/without vegetables (*n* = 59, 95.2%) and fruit with cereal/milk products (*n* = 34, 91.9%) as the most represented categories. No added salt claim was found on 27.2% (*n* = 52), with vegetables purée (*n* = 3, 100%) and savoury puréed meals (*n* = 12, 70.6%) as the most represented categories. In total, 34.0% of the baby foods (*n* = 65) contained at least one sugar-contributing ingredient. Added sugars (identified by the ingredient list as sugars, fruit juice/concentrates) were found in 13.9% (*n* = 27) and 15.0% (*n* = 29), respectively (Table 4).

Additionally, it was found that 17.3% (*n* = 23) of these products that contained sugar (*n* = 2) or concentrated fruit juice (*n* = 21) in the ingredient list had the claim “No added sugar”. 

### 3.4. Processing Level

Our results show that 61.8% (*n* = 118) of the CACFs were UP and 36.6% (*n* = 70) were MP and when comparing the nutritional composition between NOVA groups, UP were higher in energy density, proteins, total fat, saturated fatty acids, and carbohydrates compared to processed and minimally processed foods (Table 5). 

Overall, the median energy density of the UPFs was 383 kJ (91.0 kcal), with 3.3% of total energy from proteins, 16.5% from carbohydrates, and 2.5% from lipids. Per 100 g, total sugars were significantly lower (*p ≤* 0.001) when compared with MP foods as well as between processed and MP foods. There were significant differences between medians for energy (kcal, kJ) and all the nutrient content (*p* < 0.001) between each of the NOVA groups (Table 5).

**Figure 4:**
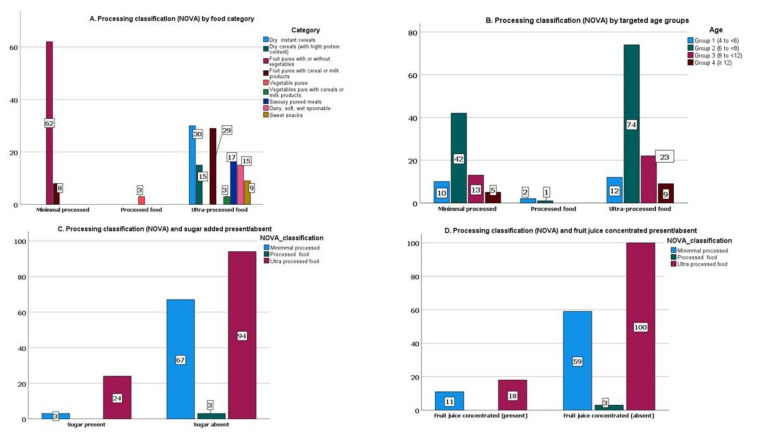
(**A**) Processing classification (NOVA) by food category (*n*); (**B**) processing classification (NOVA) by targeted age groups (*n*); (**C**) processing classification (NOVA) and added sugars (sugar) (present/absent) in the ingredient list (*n*); (**D**) processing classification (NOVA) and added sugars (fruit juice concentrated) (present/absent) in the ingredient list (*n*).

Concerning processing classification and food category, the highest proportion of minimally processed foods was observed for fruit purée with/without vegetables with 88.6% (*n* = 62). Dry instant cereals at 25.4% (*n* = 30), fruit purée with cereal/milk at 24.6% (*n* = 29), and savoury puréed meals at 14.4% (*n* = 17) represent the highest percentage of UP foods, respectively (Figure 4A).

Regarding the recommended age for consumption, our study revealed that 57.0% (*n* = 109) of the analysed products classified as UP foods were indicated for children under 12 months (Groups 1 to 3) (Figure 4B). 

In the current study, the presence of sugar and fruit juice concentrated was found in 27.1% (*n* = 32) of the UP foods and 20.0% (*n* = 14) of the MP foods (Figure 4C,D).

## 4. Discussion

This study provides some important information on the nutrient profile and level of processing of baby foods available in the market target to children up to 36 months.

Analysing the energy content, our results are similar to Gómez Martin et al., a study that identified complementary food, vegetable, legumes, and infant purée with lower energy content. Grammatikaki et al. found on average the highest content for energy in baby biscuits and rusks (sweet snacks) [18,39].

Concerning the energy density (kcal/100 g) by food category, several CACFs indicate for children up to 6 months, such as the case of vegetable purée and vegetables with cereals or milk products, provide a lower energy density, less than 60 kcal/100 g, which is the minimum energy density adopted by the NPM of WHO/Europe for CACFs [8]. Low energy density can be a problem because the small stomachs of babies and young children limit them to relatively small amounts of consumption at mealtimes. Conversely, some products in the sweet snacks category that are suitable for children above 6 months have a high energy density, thus enhancing the risk of excessive energy intake and unfavourable gain in body mass [40].

As the European Directive 2006/125/EC does not establish any requirements regarding energy density, all CACFs effectively comply with this directive [9].

Regarding dry products, the guidelines for formulated complementary foods, WHO-Codex CAC/GL 8-1991, indicate that energy density should be a minimum of 4 kcal/g (400 kcal/100 g) on a dry-weight basis [41]. The median energy density for dry cereals with high protein are in accordance with the Codex recommendation for this food category [41].

The highest protein contents in dry instant cereals and dry cereals with high protein, 10.0 g/100 g and 13.6 g/100 g, respectively, are in line with Grammatikaki et al. study, which reports a high-protein content in baby cereals, 10.5 g/100 g, respectively [18]. 

According to our data, the categories fruit purée with/without vegetables and fruit purée with cereal/milk presented higher content of carbohydrates than the categories vegetable purées, vegetable purée with cereal, and savoury meals; these findings are in line with those in the Garcia et al. study [14].

The average energy contribution from total sugars was above 75% in fruit purée (with/without vegetables). Similarly, the Hutchinson et al. study found added sugars present in a considerable percentage of fruit purées, with the mean for each country ranging between 72–79% [15].

For the sweet snacks category, the products with more than 15% of energy from sugars, these products should not be marked as suitable for IYC according to the proposed WHO criteria [8]. These results could be considered high according to the WHO recommendations to limit free sugar consumption to 5% or 10% of their overall energy intake and EFSA average requirements sets for energy for IYC [11,42].

These results are of great concern, as food preferences established in infancy persist over time [1]. Persistent exposure to foods with high levels of sugar is linked to an increase in the development of dental caries and metabolic diseases in childhood and later life [43,44].

In Portugal, children under 5 years old had the highest energy contribution from total sugars (28 E%). The main food sources contributing to added and free sugars intake were milk, yoghurts, fruit, and infant formula and, in children above 5 years, mostly processed foods, such as yoghurts, breakfast cereals, sweets, cakes, and soft drinks [13].

According to the recommendations of health organisations and experts, it is important to avoid adding salt to foods for IYC and limited thereafter because of the health risks and as a form of promoting healthier eating habits [45,46]. For the adult population, WHO and EFSA recommend a target sodium intake of 2 g per day (equivalent to 5 g per day of salt). For children, this threshold should be adjusted to a lower value due to reduced energy intake. As some foods have sodium naturally present, the study was not able to discriminate if and when salt had been added [45,47]. 

Our results converge with the results described by Maalouf et al. and Padarath et al., which found only a few of the CACFs presenting an excess of sodium content or containing an added salt [48,49].

The limit of the European Commission Directive for sodium is 200 mg/100 g or 200 mg/100 kcal or 100 mg/100 g or 100 mg/100 kcal for cereal products, and in our study, a small number of products exceeded the sodium thresholds [9]. 

The proportion of products meeting the nutritional requirements of the NPM observed in our study is similar to the Pace pilot study conducted in Malta to assess the nutritional characteristics of CACFs according to the NPM proposed by the WHO/Europe. In the pilot study, only 36% of the 243 food products tested met the nutritional requirements used by this model [50]. 

Regarding the presence of claims, the NPM for CACFs indicates no claims (compositional, health, or marketing) are accepted on the product’s packaging or related marketing materials [8]. In addition, Codex guidelines state that foods for IYC must not carry nutrition or health claims [51]. The reason for this is that claims can confuse consumers and/or compromise breastfeeding or complementary feeding practices [7].

Our results for visual aspects of baby food labels related to inadequate promotion are in concordance with the WHO study, which revealed a proportion of products with cartoons on the packaging varied from 16% in Israel to 53% in Bulgaria [52]. 

In our study, the presence of sugar-contributing ingredients complies with the European Commission directive and EFSA definition of added sugars (fruit juice is excluded); nevertheless, these ingredients will also account for the total and free sugar content of these foods [9,15,53]. Our results are in line with those by Grammatikaki et al. that reported 38.5% of the baby foods contained at least one sugar-contributing ingredient in the form of added sugars, free sugars, and fruit and vegetable purées/powders. [18]. Similarly, Padarath et al. found that 34.0% of CACFs available included several free sugars (e.g., sugars, maltodextrin, fruit juice, fruit juice concentrates, and glucose) [49]. 

Our results show that more than 50% of the CACFs were UP. Comparable results were reported in the research of Araujo et al., in which 33.6% were MP foods, 10.2% were processed, and 56.1% were UP foods [33]. 

Furthermore, in our study, UP foods showed a total sugar content per 100 g that was significantly lower and a sodium content significantly higher compared to MP foods, and the same was found between processed and MP foods. McCann et al. also found the same unexpected results for the content of total sugars and sodium in UP foods in comparison to processed and MP foods [17]. As the MP foods group consists of fruit purée with/without vegetables 88.6% (*n* = 62) and fruit purée with cereals/milk 24.6% (*n* = 8), which have in common the use of certain ingredients, this may explain the higher sugar results observed. The sodium result in the processed food group can be attributed to the group being composed of vegetable purées (*n* = 3), which have a higher sodium content compared to fruit purées. 

Da Rocha et al. found in their study that 79% of the baby foods were UP foods. The difference observed compared with our results (61.8%, *n* = 118) might be explained by the fact their study considered infant and follow-up formulas (*n* = 31, 33%), and our analysis included the food category fruit purée with cereals/milk, which had 15.8% (*n* = 29) of foods classified as UP due to the presence of additives in the ingredient list or the manufactured process [16]. 

Regarding the recommended age for consumption, our study revealed an early introduction of UP foods. Recognizing the first 1000 days as a crucial period for preventing childhood obesity, the adoption from the early age of dietary patterns that includes foods with sugars as added ingredients or high in UP foods can have implications for child health and contribute significantly to the increase of childhood obesity and chronic diseases in later life [19,54,55]. In its context, the European Childhood Obesity Group highlighted the negative effects of high consumption of UPFs by children and called for restrictions [32].

Various studies have revealed that the main consumers of UP foods are children and adolescents, with caloric intake from UP foods ranging from 67% in the USA in 2018 in children aged 2–19 years [56] to 45.5% in France by the analysis of data from the INCA3 2014–2015 study (children aged 1–10 years) [57]. In Brazil, the proportion of total UP foods energy consumption was reported as 42.0% ± 8.7 at ages 3–4 years and 47.8% ± 8.9 at ages 6–8 years [58].

The consumption of processed and UP foods by children is indicated as a factor that can decrease the quality of children’s diets, considering that many are processed and UP foods that have higher amounts of nutrients of concern, such as sugar, trans fat, and sodium, compared to unprocessed or minimally processed foods [59]. This reveals the need to establish guidelines for the infant-child food industry to reduce processing levels in the manufacture of foods [32,59].

In the current study, UP foods that contained at least one sugar-contributing ingredient, sugar was identified in 53.3% (*n* = 24) of UP foods. On the other hand, MP foods that have at least one sugar-contributing ingredient, such as fruit juice or concentrates, were found in 91.7% (*n* = 11) of MP foods. Our results are consistent with the Grammatikaki et al. study, which found in 60% of MP foods contained free sugars (e.g., fruit juices or fruit juices concentrates) [18].

In the future, as part of the strategy of nutritional reformulation of food products with excessive content of fat, salt, sugar, or energy, the present analysis may be replicated to monitor the nutritional composition of CACFs targeted to IYC under 3 years of age in Portugal.

Strengths and limitations could be identified. An important strength of this study lies in providing valuable insight into CACFs at the national level and reinforces that action is needed to establish marketing and product guidelines and inform policymakers to align with health priorities important to IYC. Furthermore, this study also classifies CACFs by the NOVA classification, providing additional insight into these products and could contribute to the limited research available in this area. Furthermore, our results also constitute an important challenge for policymakers to consider lowering the sugar content in CACFs via health taxes, reformulation programmes, and food marketing restrictions [60]. In addition, it is advisable to undertake development of restrictive regulations for CACFs labelling to assist caregivers in making healthier choices. The inclusion on labels of information on added and intrinsic sugars content should be considered [8].

Due to the inherent limitations regarding the sugars labelling of CACFs, as sugars naturally occurring in milk and fruit are not distinguished from added sugars, it was not possible to collect data on the contribution of added sugars to total energy or total carbohydrates content.

## 5. Conclusions

This study is an overview of the nutrient profile, promotional aspects of product packaging, and level of processing of CACFs and contributes with the expertise to the implementation of the WHO’s guidance of these foods. 

Our results indicate a concern related to the sugar content of several categories of CACF, as it is higher than recommended and contradicts the guidelines for restricting sugar intake in infants and young children. Furthermore, our study indicates that an early inclusion of UP foods in IYC diet is probable, with more than 50% of products destined for children under 12 months being classified as UP.

Proper nutrition during infancy and early childhood is crucial to support the growth, health, and development of children. The results of this study emphasise the importance of implementing the WHO guidelines on the assessment of commercially available foods for IYC as a strategy to improve complementary feeding practices and the importance of assessing the food processing characteristics of processed foods and beverages targeted to IYC under 3 years of age to assist parents and health professionals with the correct information when selecting complementary and infant foods.

These findings endorse the importance of innovative interventions in the field of consumer education to decrease UP foods consumption and encourage the consumption of natural and MP foods. 

This study also provides evidence to operationalize the implementation at the national level of NPM proposed by WHO to facilitate decisions on what foods are inappropriate for promotion for IYC under 36 months of age and to policymakers to update regulation and guide product reformulation concerning the sugar content of CACFs.

The data framed by WHO recommendations have essential applications in nutrition research, dietary counselling, and public health practice. The WHO recommendations are crucial to understand concerns detected in multicentre studies at the international or national level and identify sub-populations at risk due to typical dietary patterns or increased physiological needs.

## Figures and Tables

**Table 1:** Distribution of CACFs according to food category, processing level (NOVA), and age group.

Characteristic	Classification	Number (*n*)	Percentage (%)
Product/Food category	Dry instant cereals	30	15.7
	Dry cereals (with high-protein food (added)-powder milk or whey)	15	7.9
	Fruit purée (with/without vegetables)	62	32.5
	Fruit purée with cereal/milk	37	19.4
	Vegetable purée	3	1.6
	Vegetables purée with cereal, soft, wet, spoonable	3	1.6
	Savoury puréed meals	17	8.9
	Dairy, soft, wet, spoonable	15	7.9
	Sweet snacks	9	4.7
Processing level (NOVA)	Minimally processed	70	36.6
	Processed	3	1.6
	Ultra-processed	118	61.8
Age group (months)	Group 1 (4 to <6)	24	12.6
	Group 2 (6 to <8)	117	61.3
	Group 3 (8 to <12)	36	18.8
	Group 4 (≥ 12)	14	7.3

**Table 2:** Nutritional composition per 100 g for CACFs by food category, considered in the study.

	Dry Instant Cereals	Dry Cereals (with High-Protein Food)	Fruit Purée (with/without Vegetables);	Fruit Purée with Cereal/Milk	Vegetable Purees	Vegetables with Cereal, Soft, Wet Spoonable	Savoury Puréed Meals	Dairy, Soft, Wet, Spoonable	Sweet Snacks	*p* *
Energy (kJ)	1651 (1617–1696)	1739 (398–1781)	243 (220–274)	271 (244–305)	105 (81.0–†)	223 (195–†)	254 (236–296)	339 (276–383)	1810 (1787–1903)	<0.001
SD	57	624	41	49	47	38	39	50	60	
Protein (g)	10.0 (7.9–12.0)	13.6 (2.9–16.5)	0.4 (0.3–0.7)	0.7 (0.4–1.0)	0.8 (0.3–†)	1.3 (0.7–†)	2.7 (2.4–2.9)	3.0 (2.7–3.1)	8.7 (6.7–9.7)	<0.001
SD	3.8	5.8	0.2	0.7	0.3	0.8	0.4	0.5	2.8	
Total fat (g)	2.3 (1.8–4.6)	9.5 (3.0–11.0)	0.2 (0.1–0.2)	0.3 (0.2–1.0)	0.3 (0.2–†)	1.1 (1.0–†)	1.8 (1.7–2.2)	2.8 (2.3–3.2)	10.0 (8.6–13.5)	<0.001
SD	2.8	3.9	0.2	1.4	0.7	0.7	0.4	0.6	2.4	
Saturated fatty acids (g)	0.5 (0.3–1.5)	1.8 (1.3–2.5)	0.0 (0.0–0.6)	0.0 (0.0–0.3)	0.0 (0.0–†)	0.1 (0.0–†)	0.4 (0.3–0.6)	1.5 (1.3–2.0)	1.3 (1.3–3.3)	<0.001
SD	1.2	1.3	0.2	0.7	0.2	0.4	0.2	0.4	1.9	
Carbohydrate (g)	76.7 (75.0–82.0)	63.0 (14.0–66.4)	12.9 (11.3–14.1)	13.0 (12.0–15.0)	4.1 (3.5–†)	8.0 (8.0–†)	7.60 (6.6–9.3)	11.5 (8.8–12.2)	76.0 (75.0–76.0)	<0.001
SD	7.3	24.8	2.3	1.9	1.2	0.3	1.6	2.3	1.9	
Total sugars (g)	20.0 (1.5–26,2)	27.2(8.0–31.1)	11.0 (9.1–12.1)	10.7 (9,6–12.0)	1.5 (1.3–†)	2.9 (2.0–†)	1.3 (1.2–2.4)	7.2 (5.0–8.1)	12.0 (2.8–24.0)	<0.001
SD	12.7	10.7	2.2	1.7	0.5	0.7	0.9	2.0	11.0	
Sodium (mg)	16.0 (4.0–45.0)	80.0 (36.0–108)	4.00 (2.0–16.0)	4.00 (0.0–16.5)	28.0 (25.2–†)	16.0 (12.0–†)	32.0 (20.0–78.0)	36.0 (30.0–52.0)	32.0 (8.0–80.0)	<0.001
SD	54.0	49.9	14.4	11.6	17.0	8.3	40.0	14.7	68.9	

Values are expressed as median (25th–75th percentile); SD, standard deviation; * *p*-value obtained with the Kruskal–Wallis test for independent samples with multiple pairwise comparisons (food categories); † Not calculated (small sample size, *n* = 3).

**Table 3:** Distribution of claim type and visual information for CACFs considered in the present study.

Characteristic	Sub-Category or Classification	Number (*n*)	Percentage (%)
Nutrition/Composition Claim	Yes	185	96.9
	No	6	3.1
Health Claim	Yes	17	9.0
	No	174	91.0
Claim type	**Composition claim**		
	No added preservatives	57	29.8
	Gluten-free	64	33.5
	Organic food	47	24.6
	Egg-free	15	7.9
	Dairy-free	18	9.4
	Nutrition claim		
	No added sugar	133	69.6
	No added salt	52	27.2
	Contains vitamin C	26	13.6
	Contains iron	19	9.9
	Contains calcium	27	14.1
	Contains vitamin E	3	1.6
	Contains multiple vitamins	16	8.4
	Contains multiple minerals	13	6.8
	Contains dietary fiber	8	4.2
	**Health claim**		
	Nutritionally balanced/provides good nutrition to children	15	7.9
	Cognitive ability	2	1.0
Visual Information (cartons, pictures)	Yes	74	39.0
	No	117	61.0

**Table 4:** Description of CACFs according to the presence of claims and presence of sugar-contributing ingredients (number (*n*) and percentage (%)).

	Dry Instant Cereals	Dry Cereals (with High-Protein Food)	Fruit Purée (with/without Vegetables)	Fruit Purée with Cereal/Milk	Vegetable Purée	Vegetables Purée with Cereal, Soft, Wet, Spoonable	Savoury Puréed Meals	Dairy, Soft, Wet, Spoonable	Sweet Snacks	Total ^1^
**Total**	30 (15.7%)	15 (7.9%)	62 (32.5%)	37 (19.4%)	3 (1.6%)	3 (1.6%)	17 (8.9%)	15 (7.9%)	9 (4.7%)	193 (100%)
**Composition Claim** No added preservatives ^(a)^	2 (6.7%)	1 (6.7%)	28 (45.2%)	7 (19.9%)	0 (0.0%)	0 (0.0%)	12 (70.6%)	7 (46.7%)	0 (0.0%)	57 (29.8%)
Gluten-free ^(b)^	6 (20.0%)	3 (20.0%)	29 (46.8%)	10 (27.0%)	3 (100.0%)	1 (33.3%)	4 (23.5%)	7 (46.7%)	1 (11.1%)	64 (33.5%)
Organic food ^(a)^	1 (3.3%)	0 (0.0%)	17 (27.4%)	16 (43.2%)	0 (0.0%)	3 (100.0%)	5 (29.4%)	4 (26.7%)	1 (11.1%)	47 (24.6%)
**Nutrition Claim** No added sugar (^a)^	16 (53.3%)	5 (33.3%)	59 (95.2%)	34 (91.9%)	0 (0.0%)	0 (0.0%)	5 (29.4%)	10 (66.7%)	4 (44.4%)	133 (69.6%)
No added salt ^(a)^	13 (43.3%)	5 (33.3%)	10 (16.1%)	3 (8.1%)	3 (100.0%)	2 (66.7%)	12 (70.6%)	1 (6.7%)	3 (33.3%)	52 (27.2%)
**Health Claim** Nutritionally balanced/provides good nutrition to children	0 (0.0%)	1 (6.7)%	0 (0.0%)	3 (8.1%)	0 (0.0%)	0 (0.0%)	0 (0.0%)	10 (66.7%)	1 (11.1%)	15 (7.9%)
**Ingredients (added sugars)** Sugar ^(a)^	9 (30.0%)	5 (33.0%)	3 (4.8%)	0 (0.0%)	0 (0.0%)	0 (0.0%)	0 (0.0%)	5 (33.0%)	5 (55.6%)	27 (13.9%)
Fruit juice concentrated ^(c)^	2 (6.7%)	3 (20.0%)	11 (17.7%)	11 (29.7%)	0 (0.0%)	0 (0.0%)	0 (0.0%)	2 (13.3%)	0 (0.0%)	29 (15.0%)
Other added sugars ^(d), (e), (f)^	5 (16.6%)	0 (0.0%)	0 (0.0%)	3 (8.1%)	0 (0.0%)	0 (0.0%)	0 (0.0%)	0 (0.0%)	1 (11.1%)	9 (4.7%)

χ^2^ test, ^(a)^ (*p* < 0.001), ^(b)^ (*p* = 0.014), ^(c)^ (*p* = 0.079), and ^(d)^ (*p* > 0.05) for syrup, dextrose, sucrose, malt extract; ^(e)^ *p* = 0.038, for honey; ^(f)^ *p* = Not calculated because variables is a constant for malted barley extract, molasses, maltose, fructose, glucose, trehalose, galactose; ^1^ Total percentage for each claims type and presence of sugar-contributing ingredients is in relation to the total products analysed (*n* = 193).

**Table 5:** Nutrition information per 100 g across processing classification (NOVA) for CACFs considered in the study.

	Minimally Processed	Processed	Ultra-Processed	*p* *
Energy (kJ)	245 (222–272) ^a,c^	105 (81.0–†) ^a^	383 (276–1681) ^b^	<0.001
SD	39	47	708	
Energy (kcal)	58.0 (52.5–64.5) ^a,c^	24.9 (19.0–†) ^a^	91.0 (66.0–397.3) ^b^	<0.001
SD	9.0	11.3	167.4	
Protein (g)	0.4 (0.3–0.7) ^a^	0.8 (0.3–†) ^a,c^	3.0 (1.9–10.0) ^b,c^	<0.001
SD	0.2	0.3	5.0	
Total fat (g)	0.2 (0.1–0.2) ^a^	0.3 (0.2–†) ^a,c^	2.3 (1.1–4.0) ^b,c^	<0.001
SD	0.2	0.7	3.6	
Saturated fatty acids (g)	0.0 (0.0–0.0) ^a^	0.0 (0.0–†) ^a,c^	0.7(2.9–1.8) ^b,c^	<0.001
SD	0.2	0.2	1.2	
Carbohydrate (g)	12.9 (11.4–14.0) ^b^	4.1 (3.5–†) ^a^	15.0 (11.2–75.0) ^c^	<0.001
SD	2.2	1.2	32.0	
Total sugars (g)	11.0 (9.5–12.1) ^b,c^	1.5 (1.3–†) ^a^	9.0 (2.9–19.0) ^a,c^	0.040
SD	2.1	0.5	10.4	
Sodium (mg)	4.0 (0.4–12.0) ^a^	25.2 (0.56–†) ^a,c^	24.6 (8.0–52.0) ^b,c^	<0.001
SD	14.0	15.0	46.0	

Values are expressed as median (25th–75th percentile); * *p*-value obtained with Kruskal–Wallis test for independent samples with multiple pairwise comparisons (processing classification). For each processing level; different lowercase letters in the same row indicate differences among processing (Kruskal–Wallis non-parametric one-way ANOVA for independent samples with multiple pairwise comparisons), † Not calculated (small sample size, *n* = 3); SD, standard deviation.

## Data Availability

The data presented in this study are available on request from the corresponding author.

## Appendix A

**Table A1:** Information on the coded variables that were subject to analysis.

Coded Variables	Description
Basic information	Brand name, product name, food type, ingredients, recommend age, serving size
Product category	Products were classified according to the NPM proposed by WHO/Europe: Dry instant cereals; dry cereals (with high-protein food (added)-powder milk or whey); fruit purée (with/without vegetables); vegetable purées; fruit purée with cereal/milk; vegetables with cereal, soft, wet, spoonable; savoury puréed meals; dairy, soft, wet, spoonable; sweet snacks.
Nutritional information	Nutritional content (total energy (kJ/kcal), protein (g), carbohydrates (g), total sugars (g), fat (g), saturated fatty acids (g), and sodium (mg). All the nutritional analysis was per 100 g product.
Details on the presence of added sugars	For these analyses, added sugars were classified as fruit juice whether whole, concentrated, or powdered; sugar; sucrose; dextrose; fructose; maltose; any syrup; honey; barley malt/malted barley/malt extract; molasses; and artificial or natural zero/low-calorie sweeteners.
Composition, nutrition, and health claims	**Composition claim**: No added preservatives, organic food, egg-free, dairy-claims free, gluten-free. **Nutrition claim**: Contains calcium, contains iron, contains vitamin C, contains dietary fiber, contains multiple vitamins, no added sugar, no added salt, contains vitamin E, contains multiple minerals. **Health claims**: Provides good nutrition to children, nutritionally balanced, cognitive ability.
Visual information	Presence of cartoons, pictures of infants/young children, pictures of mothers, pictures of bottles/teats.
